# Identification and Comprehensive Genome-Wide Analysis of Glutathione S-Transferase Gene Family in Sweet Cherry (*Prunus avium*) and Their Expression Profiling Reveals a Likely Role in Anthocyanin Accumulation

**DOI:** 10.3389/fpls.2022.938800

**Published:** 2022-07-12

**Authors:** Irfan Ali Sabir, Muhammad Aamir Manzoor, Iftikhar Hussain Shah, Xunju Liu, Songtao Jiu, Jiyuan Wang, Pravej Alam, Muhammad Abdullah, Caixi Zhang

**Affiliations:** ^1^Department of Plant Science, School of Agriculture and Biology, Shanghai Jiao Tong University, Shanghai, China; ^2^School of Life Sciences, Anhui Agricultural University, Hefei, China; ^3^Department of Biology, College of Science and Humanities, Prince Sattam Bin Abdulaziz University, Al-Kharj, Saudi Arabia

**Keywords:** Glutathione S-transferases, *Prunus avium*, phylogeny, expression analysis, anthocyanin

## Abstract

Glutathione S-transferases (GSTs) in plants are multipurpose enzymes that are involved in growth and development and anthocyanins transportation. However, members of the GST gene family were not identified in sweet cherry (*Prunus avium*). To identify the GST genes in sweet cherry, a genome-wide analysis was conducted. In this study, we identified 67 GST genes in *P. avium* genome and nomenclature according to chromosomal distribution. Phylogenetic tree analysis revealed that *PavGST* genes were classified into seven chief subfamily: TCHQD, Theta, Phi, Zeta, Lambda, DHAR, and Tau. The majority of the *PavGST* genes had a relatively well-maintained exon–intron and motif arrangement within the same group, according to gene structure and motif analyses. Gene structure (introns-exons) and conserved motif analysis revealed that the majority of the *PavGST* genes showed a relatively well-maintained motif and exons–introns configuration within the same group. The chromosomal localization, GO enrichment annotation, subcellular localization, syntenic relationship, Ka/Ks analysis, and molecular characteristics were accomplished using various bioinformatics tools. Mode of gene duplication showed that dispersed duplication might play a key role in the expansion of *PavGST* gene family. Promoter regions of *PavGST* genes contain numerous *cis*-regulatory components, which are involved in multiple stress responses, such as abiotic stress and phytohormones responsive factors. Furthermore, the expression profile of sweet cherry *PavGSTs* showed significant results under LED treatment. Our findings provide the groundwork for future research into induced LED anthocyanin and antioxidants deposition in sweet cherries.

## Introduction

Many physiological and metabolic processes are dependent on transcriptional regulation of gene expression in plants, particularly response to various environmental stimulation and stressors like drought, salt, and cold along with defensive responses toward pathogenic organisms (Qu and Zhu, 2006). The action of these transcription factors (TFs) can retain, improve, or suppress gene expression at the molecular level. The functional regulation of these TFs represents a dynamic mechanism that assists higher plants to adapt to environmental changes, as well as other developmental and genetic responses (Benfey and Weigel, 2001; Carroll, 2005). Mainly, variations in TFs activity or quantitative levels are responsible for regulating gene expression. Based on their conserved domains, more than 84 families of TFs have been identified so far (Pérez-Rodríguez et al., 2010). The Glutathione S-transferases (GSTs) TF family was first identified in maize (Frear and Swanson, 1970), and is responsible for the transport and metabolism of secondary compounds, including porphyrins, flavonoids, and anthocyanins (Dixon et al., 2010). GSTs are quite a vast, old, and diversified family of multifunctional proteins present in virtually all aerobic species, including humans. In many plants, GSTs are found in the cytoplasm, where they might be found as monomers, or heterodimers of 23–30 kDa, depending on the type of GST. Each subunit has a glutathione binding (G-site) at the N-terminus domain and a C-terminal domain that defines second-substrate specificity (C-site; Edwards et al., 2000).

Plant GSTs are categorized into 11 classes according to dynamic site residues, protein sequence resemblance, and genetic association: Tau, Zeta, Phi, Lambda, Theta, TCHQD (tetrachlorohydroquinone dehalogenase), DHAR (dehydroascorbate reductase), mPGES2 (microsomal prostaglandin -e -synthase type 2), EF1G (elongation factor 1; Sheehan et al., 2001). TCHQD, Tau, Phi, and Lambda are plant-specific GSTs between all these classes. Furthermore, *In silico* investigations have recently confirmed that there are 79 genes in *Oryza sativa* (rice; Jain et al., 2010), 53 *Arabidopsis thaliana* (Sappl et al., 2009), 90 in *Solanum lycopersicum* (tomato; Islam et al., 2017), 23 in *Citrus X sinensis* (sweet oranges; Licciardello et al., 2014), 85 in *Piper nigrum* (black paper; Islam et al., 2019), 42 in *Zea mays* (maize), 25 in *Glycine max* (soybean; McGonigle et al., 2000), 52 in *Malus domestica* (apple) and 50 in *Prunus persica* (peach; Fang et al., 2020).

Flavonoids are secondary metabolites found in virtually all higher plants and support a variety of biological activities. They are the primary mediators of plant auxin transport and might be deployed to protect plants from biotic and abiotic stressors (Winkel-Shirley, 2001). Anthocyanin is a kind of flavonoid found in a diverse array of higher plants. They are important for attracting seed dispersers (Vogt et al., 1994) and UV protection (Sarma and Sharma, 1999). Anthocyanin is a kind of water-soluble pigment found in flavonoid compounds that are found in a variety of plants. The pH value of vacuoles influences the development of color (Jackson et al., 1992; Steyn et al., 2002). Anthocyanins work as a ripening indicator for many species, suggesting that fruits with more anthocyanin content have better market value and health advantages (Boyer and Liu, 2004). The presence of anthocyanin not only enhances the fruit quality but also has economic consequences. The cytosol produces anthocyanins, which are then transferred to the vacuole for storage. The mechanism of transporting anthocyanins is not as well known as the method of synthesis. Recently, three distinct hypotheses regarding the transit of anthocyanin to the vacuole have been proposed: GST-mediated pathway, membrane transporters, and vesicle trafficking, and all of these functioned in a well-collaborated way (Zhao, 2015). Anthocyanin is transported from endoplasmic reticulum (ER) to the vacuole through GSTs (Dixon et al., 2010; Zhu et al., 2016), which are key non-catalytic carrier proteins for anthocyanin absorption by vacuoles (Kitamura et al., 2004; Zhu et al., 2016). Furthermore, functional investigations have shown that particular plant GSTs are required for anthocyanin and proanthocyanidin vacuolar deposition. The role of these GSTs in vacuolar anthocyanin transportation was initially discovered in the maize bronze-2 (bz2, GST-like protein) mutant (Maars et al., 1995), which exhibited apparent pigmentation deficit. GSTs with comparable roles for anthocyanin have now been discovered in a variety of plant species, including *VviGST1* and *VviGST4* in *Vitis vinifera* (Conn et al., 2008), *FvRAP* in *Fragaria vesca (Luo et al., 2018)*, *AcGST1* in *Actinidia deliciosa* (Liu et al., 2019), *MdGSTF6* in *M. domestica* (Jiang et al., 2019), and *PpGST1* in *P. persica* (Zhao et al., 2020) have been described as crucial for floral or fruit pigmentation.

Sweet cherries (*Prunus avium* L.) are nutrient-dense fruit that is high in anthocyanins, a kind of dietary flavonoids responsible for its red pigmentation. Anthocyanins are associated with both defensive and physiological processes, as well as assisting with sweet cherry sensory attributes. Additionally, anthocyanins may be responsible for the health advantages associated with sweet cherry consumption. Anthocyanin transportation is mediated by GST genes in plants. Moreover, a genome-wide investigation of GST genes was conducted in sweet cherry because of the reported involvement of GSTs in mediating anthocyanin accumulation (Maars et al., 1995; Mueller et al., 2000; Sun et al., 2012; Pérez-Díaz et al., 2016). Each member’s physiochemical characteristics, subcellular localization, chromosomal position, evolutionary linkage, and gene duplication events have all been studied in more detail. In addition, the expression of sweet cherry GSTs upon LED treatment was investigated. Our findings provide the groundwork for future research into induced LED anthocyanin deposition in sweet cherry.

## Materials and Methods

### Physico-Chemical Characterization and Identification of Glutathione S-Transferase Genes in *Prunus avium*

The *P. avium* genome sequences were obtained for GST gene annotation and identification: from the Genome Database for Rosaceae (GDR^1^; Verde et al., 2013; Jung et al., 2014; Daccord et al., 2017; Shirasawa et al., 2017; Aamir et al., 2020). To further verify the conserved domains like GST_C (PF00043) and GST_N (PF02798). Initially, the protein sequence alignment for GST C (PF00043), GST N (PF02798), (Pfam GST domain) was obtained in Stockholm format, and Hmmbuild was used to create a model from that alignment. The Hmmsearch program was being utilized to find all potential genes through the whole-genome database of *P. avium* with BLASTP (E < 1e^––5^). Furthermore, 57 protein sequences of *A. thaliana* were utilized as a query to retrieve 67 potential GST protein sequences of the sweet cherry genome. The sequences of all GST proteins were aligned, and repetitive genes were eliminated. All GST genes were then validated through InterProScan^2^ (Zdobnov and Apweiler, 2001; Finn, 2006; Letunic et al., 2012; Manzoor et al., 2021a), SMART^3^ (Letunic et al., 2012) and Pfam database^4^ (Finn, 2006). The ExPASY server^5^ was used to anticipate molecular properties (length of amino acid, IP (isoelectric point) and weight) while, WoLF PSOPT II^6^ was used to determine the subcellular localization (Horton et al., 2007; Abdullah et al., 2018a; Cao et al., 2018; Manzoor et al., 2021a).

### Phylogeny Analysis of Glutathione S-Transferase Genes Family

ClustalX software was used to align all GST full-length protein sequences with default parameters (1000 bootstrap, pairwise deletion; Thompson et al., 1997; Abdullah et al., 2018b). MEGA-X (Molecular Evolutionary Analysis) and the maximum likelihood method (ML-M) were utilized to construct the phylogenetic tree (Tamura et al., 2011). Moreover, the itols online software^7^ (Letunic and Bork, 2019; Manzoor et al., 2021b) was employed to illustrate the phylogenetic trees.

### Conserved Motif and Gene Structure Analysis

The GSDS 2.0 (Gene Structure and Display Server)^8^ was used to emphasize the important features, such as exon-intron arrangement and composition, conserved elements, and binding sites (Hu et al., 2015; Abdullah et al., 2018a; Manzoor et al., 2021c,d). The MEME software (Motif Elicitation)^9^ (Bailey et al., 2015; Sabir et al., 2022) was used to illustrate the conserved motifs.

### Conserved Domain and Chromosomal Distribution Analysis

All GST gene positions were determined through the Rosaceae Genome Database (see text footnote 1) and then visualized using Mapinspect web tool^10^ (Sneddon et al., 2012; Chen et al., 2018; Manzoor et al., 2021d). Furthermore, the Pfam database,^11^ HMMER software,^12^ and Conserved Domain Architecture Retrieval Tool (CDART) were used to identify the conserved domains of *PavGST* proteins (Geer et al., 2002; Cheng et al., 2019).

### *Cis*-Elements Analysis in Sweet Cherry

The promoter regions were discovered through the initiation codon along with 1.5 kb upstream sequences for all *PavGST* members and PlantCARE database^13^ was utilized to estimate and display all cis-elements (Lescot, 2002; Manzoor et al., 2021d).

### Gene Duplications, Collinearity Relationships, and Non-synonymous and Synonymous Mutation Rates

Collinearity analysis through BLASTP (E < 1 × 10^–5^) alongside several Rosaceae genomes was carried out using the MCScanX (Multiple Collinearity Scan Toolkit) with default parameters of OrthoMCL were used. The combination of OrthoMCL intermediate files “orthologs.txt” and “coorthologs.txt” (generated by orthomclDumpPairsFiles) was used as the whole set of ortholog pairs (Wang et al., 2012; Qiao et al., 2015; Riaz et al., 2021). MCScanX software was used to identify several types of duplications (tandem duplications, scattered, proximal, and WGD/segmental duplication) in *P. avium* while, TBtools and circos toolkits were utilized to show the collinearity correlations and gene duplications (Chen et al., 2020; Manzoor et al., 2021c). In this study, Ks (the synonymous: mutations/substitutions resulting in single amino acid alter on a given polypeptide) and Ka (non-synonymous mutation rates: mutations or substitutions that do not alter the amino acid sequence) for corresponding duplication pairs were got from the Plant Genome Duplication Database (PGDD)^14^ (Lee et al., 2013). Moreover, for computing the Ka/Ks ratio of every pair of identical genes along with multiple alignments, we used the MAFFT program and calculator^15^ (Wang et al., 2010; Qiao et al., 2019).

### Plant Material and Treatments

The sweet cherry cultivar “Van” was cultivated on the research farm of Shanghai Jiao Tong University in Shanghai, China (31.25°N, 121.48°E). The rootstock “DaQingye” (*P. pseudocerasus*) was being utilized to graft diploid cultivars. All plantations were established at a 5–6 m spacing using the same agricultural approaches. Fully mature “Van” fruits (43D) was irradiated with LED light (450 nm) for different period of time (30H, 50H, 80H, 100H). Some fresh experimental material was used for physic-chemical evaluation while the remaining experimental materials were freeze-dried and stored at –80°C till further use. Three duplicates of each treatment were utilized.

### RNA Extraction, Reverse Transcription, and Quantitative Real-Time PCR

In order to examine the qRT-PCR results, RNAiso-mate Tissue Kit was used to isolate total RNA from frozen fruit tissue (Tiangen, Beijing, China). A Nanodrop 1000 spectrophotometer was used to check the RNA’s purity and quantity (ThermoScientific, Beijing, China). A one-step RT-qPCR kit was used to reverse transcribe the RNA into the first-strand cDNA (Takara, Shanghai, China). The ABI 7500 real-time PCR detection system was used to perform quantitative RT-PCR (qRT-PCR) following the manufacturer’s instructions and an SYBR green Premix Ex TaqTM kit (Takara). As an internal control, the tubulin gene was used to normalize the gene expression data (Chun et al., 2020). The primer premier software was used to design qRT-PCR primers enlisted in Supplementary Table 7. The relative expression levels for each of the genes were measured using the 2^–Δ^ ^Δ^ *^CT^* method with three biological and technical replicates (Livak and Schmittgen, 2001; Manzoor et al., 2021a).

### Color Index Evaluation

Computation of the color index The CIELAB color system was used to measure the color of the fruit’s surface using a Minolta CR-410 colorimeter. Bayberries’ color index was determined as CIRG = (180 – h)(C + L; Carreño et al., 1995), where L is lightness and correlates to a black–white scale, h is hue angle on the color wheel, and C represents chroma, a measure of color intensity. Three distant color measurements were taken around the equator of each sweet cherry fruit, and statistical analyses were performed on the mean values for twenty fruits from each treatment.

### Anthocyanin Contents (mg CEQ/100 g) Evaluation

The flavonoids were identified using the technique used by Kim et al. (2003). 1 ml of fruit juice was mixed with 4 ml of distilled water. This was followed by the addition of a 5% sodium nitrite solution (0.3 ml) and a 10% aluminum chloride solution (0.3 ml). After 5 min of incubation at ambient temperature, 2 ml of 1M sodium hydroxide was added to the reaction mixture, followed by the addition of distilled water to bring the volume up to 10 ml. The mixture was completely vortexed, and the absorbance of the pink hue that resulted was measured at 510 nm using a spectrophotometer (Li et al., 2012). A calibration curve was developed using catechin, and the results were expressed in mg catechin equivalents. All measurements had to be taken in triplicate so that the mean values could be obtained.

### Total Antioxidants (% DPPH Inhibition)

The total antioxidant activity was determined by their ability to scavenge 2, 2-diphenyl-1-picrylhydrazyl stable radicles which were previously demonstrated by Wang et al. (1996). The absorbance was measured at 517 nm against a blank through a microplate ELISA reader (Bio Tek, United States). The following formula was used to compute the inhibition of free radicals by DPPH in the present (%).


1%=(A⁢blank-A⁢sample/A⁢blank)×100

where A blank represents the absorbance of the control reaction mixture without the sample and A sample represents the absorbance of the test substances. The IC50 values, which reflect the concentration of sweet cherry fruit extracts required to neutralize 50% of DPPH radicals, were determined by plotting the inhibition percentage against the concentrations.

## Results

### Investigation and Classification of Glutathione S-Transferases Genes in Sweet Cherry

**FIGURE 1:**
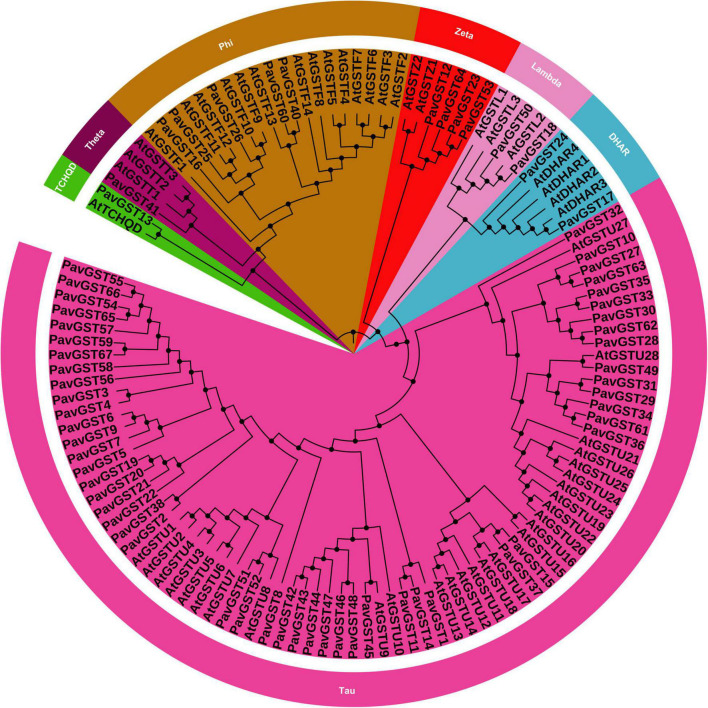
Phylogenetic tree of glutathione S-transferase (GST) gene family of *P. avium* and *Arabidopsis thaliana*. Each color representing a subfamily (TCHQD, Theta, Phi, Zeta, Lambda, DHAR and Tau) of GST genes and itol was used to construct the phylogenetic tree.

A genome-wide investigation was achieved to find all GST genes from *Prunus avium* by using a genome database. The availability of the sequenced genome of *A. thaliana* (from the Phytozome database) and Pfam-specific domains (GST C-terminus (PF00043), GST N-terminus (PF02798) permitted for the identification and prediction of all GST members in the *P. avium* genome. The BlastP and HMM were being utilized to find the GST genes in the *P. avium* genome database. The Pfam database, the Interpro tool, and the SMART database were utilized to verify the domain’s presence in all GST genes. The sweet cherry genomes were used to derive the protein and coding sequences. Finally, 67 GST genes were found and classified as *PbGST1*–67 based on their chromosomal localization or physical location (Supplementary Table 1). In sweet cherry (*PavGST*), a total of 67 GST genes were discovered (Supplementary Table 1). Additionally, the evolutionary relationship between *P. avium* and *A. thaliana* was investigated. The amino acid sequences were aligned using clustalX, and the MEGA-X program was used to construct a phylogenetic tree. Additionally, we employed two phylogenetic inference techniques, neighbor-joining (N-J) and maximum likelihood (ML), to create phylogenetic trees to validate the topologies because the N-J tree has greater bootstrap values than the other phylogenetic tree, we elected it for our future investigation (Figure 1). All discovered GSTs from *P. avium* were classified into seven subfamilies based on their bootstrap values, topography, and sequence similarity (Tau, DHAR, Lambda, Zeta, Phl, Theta, and TCHQD). Surprisingly, the Tau subfamily was the biggest, accounting for more than half of all GSTs, while the DHAR, Lambda, Zeta, Phl, Theta, and TCHQD subfamilies were quite tiny (Figure 1). For example, 52 proteins were classified as Tau among the 67 *PavGSTs*, while only one protein was identified as TCHQD (*PavGST13*) or Theta (*PavGST41*). These findings imply that members of the GST Tau subfamily proliferated more quickly in plants and presumably have a wide range of vital roles. In comparison to Arabidopsis, however, lost GST subfamilies, such as Theta and Phi, indicating that the Theta and Phi subfamilies were lost during evolution or after divergence in the last common ancestor. However, the majority of GSTs from the seven subfamilies included in the research were found to be different from Arabidopsis homologs in the phylogenetic analysis, showing that the majority of the mode of gene duplication events occurred after their divergence from Arabidopsis. Additionally, our findings showed that they might have been the consequence of gene loss or gain events that happened throughout the evolutionary process. The functional divergence occurred as a result of the accumulation and deletion of certain GSTs gene members.

### Assessment of Gene Structure and Conserved Motif of *PavGST* Genes

**FIGURE 2:**
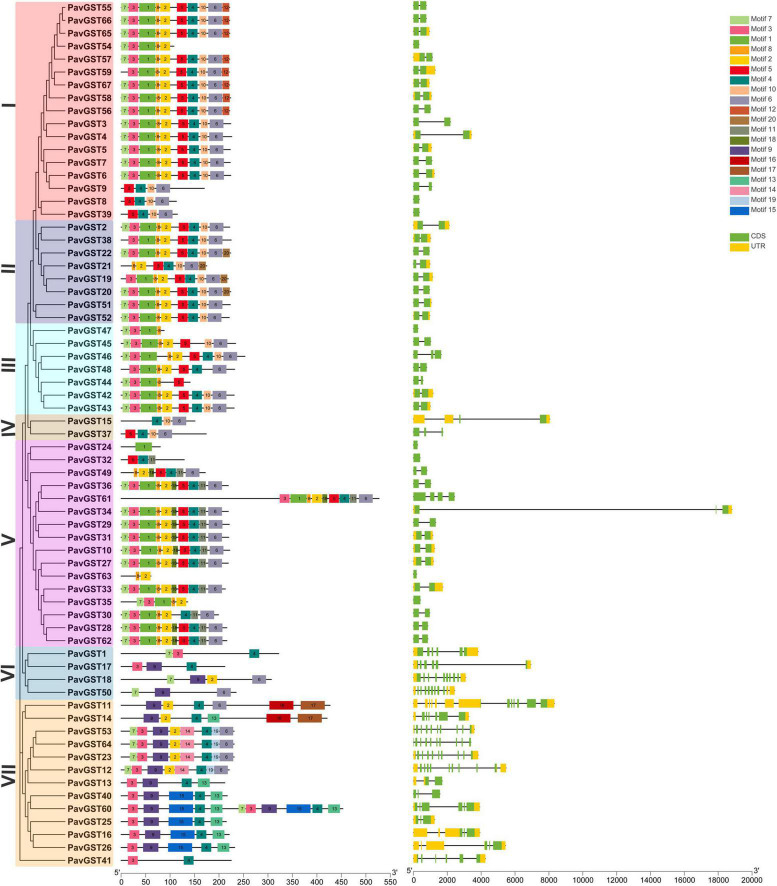
Phylogeny (left panel), conserved motifs (center panel) and intron-exons (right panel) for GST family proteins in sweet cherry were analyzed. **(A)** The motif composition of *PavGST* protein (1–20) is represented by distinct colored boxes with appropriate motif numbers and figure legends are mentioned on the top. **(B)** Phylogeny and gene structure for GST family proteins in sweet cherry. The relative position and size of the exon can be estimated using the scale at the bottom. Green boxes, black lines, and yellow boxes illustrated exons, introns, and UTR, respectively.

A phylogenetic tree was constructed to provide more insight into the structure of the 67 *PavGST* gene family members (Figure 2). We analyzed *PavGST’s* exon–intron structure and conserved motif distribution. The GST family members in each subfamily share the same conserved motifs, strengthening the phylogenetic tree conclusions. They might, though, contain diverse conserved motifs in numerous subfamilies. A total of 20 conserved motifs were investigated while using MEME software (Figure 2). Motif 3, 6, and 7 has been identified as *PavGST* conserved motifs and could be traced in all subfamilies which revealed that the addition of these three motifs (3, 6, and 7) in all subfamilies members occurred through some specific mutual evolutionary process and they have some specific function, while few subfamilies had exclusive motif formation, such as subfamilies I and II own unique motif 12 and 20, respectively, whereas motif 9 was traced only in VI and VII subfamilies which demonstrated that these two families faced some unique evolutionary processes, and these families have some unique functionalities. MEME results showed that *PavGST15* (IV subfamily) had only three motifs (Motif 4, 10, and 5). It was also identified that in the identical subfamily the motif distribution of members was extremely conservative, such as subfamily I members had motifs 7,3 1,8, 2, 5, 4, 10, and 6; while most members of subfamily II had motifs 7, 3, 1, 8, 2, 5, 4, 10, and 6 (Figure 2).

The structural components of the exons and introns of about 67 *PavGST* genes were retrieved using the Gene Structure Display Server^16^ (Figure 2). The structure of *PavGST* genes indicated a tremendous deal of diversity. The findings revealed a wide range of *PavGST* gene structures. Moreover, it was observed that the intron number varied from 1 to 12 (*PavGST12*) while the exon number varies from 1 to 10 in *PavGST* transcripts. A maximum number of exons was found in *PavGST53* and *PavGST64* (10 exons) and the minimum number of one exon was found in *PavGST54, PavGST8*, and *PavGST39* (Subfamily I) while *PavGST47* (subfamily III), *PavGST24, PavGST32, PavGST63*, and *PavGST35* (subfamily V) Transcripts (Figure 2). Intron locations are critical for proper amino acid coding and protein secondary structure; they also give an evolutionary benefit by boosting protein variety through exon shuffling and alternative splicing (Gorlova et al., 2014). Eight genes (*PavGST54, PavGST8, PavGST39, PavGST47, PavGST24, PavGST32, PavGST63*, and *PavGST35*) were identified to be intron less and hence to lack intron phase in their gene structure. The single intron was present in 35 *PavGST* members. The remainder of the genes had a variable number of introns. The relationship between the intron phase and sequence conservation at splice sites is associated with the evolution of spliceosomal introns (Long and Deutsch, 1999). Additionally, phase 0 of the intron had the maximum conservation, whereas phase 2 exhibited the lowest conservation. In case of *PavGST* classes, Subfamilies I, II, III, IV, and V exhibited peak conservation whereas subfamily VI, and VII showed a greater number of introns and mixed conservation at the splice site sequence (Figure 2).

### Examination of *Cis*-Regulating Elements in *PavGST* Genes

**FIGURE 3:**
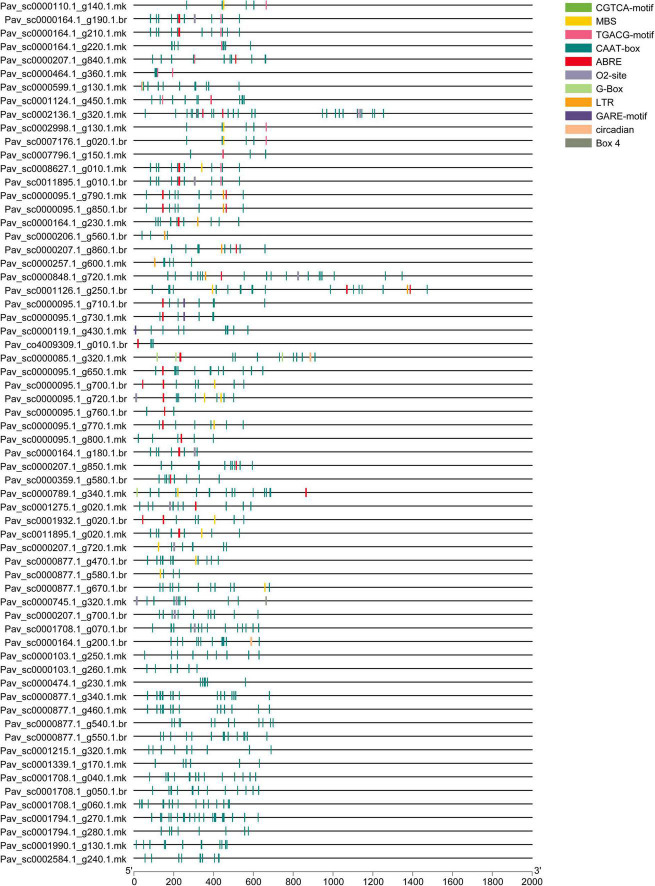
Identified *cis*-elements in the promoter regions of the *PavGST* genes. All promoter sequences (2 kb) were assessed. The *PavGST* genes are depicted on the left side of the figure. The scale bar at the bottom represents the length of promoter sequence.

Transcription factors (TFs) influence target genes both spatially and functionally *via* specialized binding of *cis*-regulatory elements found in promoters (Qiu, 2003). The genomic sequence upstream of every gene was obtained and analyzed to the PlantCARE database to investigate the *cis*-regulatory elements of the *PavGST* gene family. *Cis*-regulatory elements of *PavGST* were found to be engaged in phytohormone responses (abscisic acid, gibberellin, salicylic acid, auxin, and methyl jasmonate response elements), as well as stress responses (light, low temperature, and drought; Figure 3 and Supplementary Table 2). Several *cis*-regulatory elements were noticed to be engaged in the hormone responsiveness, such as gibberellin response element (P-box), auxin (TGA element) response elements, and promoter and enhancer regions (CAAT-box) and MeJA (CGTCA-motif, TGACG-motif). On the other hand, there were also found stress-response elements associated with ABA (ABRE), low-temperature reactivity (LTR), the MYB binding site (MBS) implicated in drought, and zein metabolism regulation (O2-site) activation (Figure 3). ABRE *cis*-elements (ABA response) were identified in 7% of *PavGST* members while 2% of total members of the MBS (MYB binding site) engaged in drought induction was found. Moreover, G-Box with 1% (Light-responsive *cis*-acting regulatory elements), Box4 with 1% (a DNA module implicated in light responsiveness), and G Box with 1% (light-responsive elements) were all discovered. The phytohormones response related *cis*-regulatory elements, such as TGACG motif (2%) and GARE-motif (1%), were also discovered, which are associated with gibberellin and MeJA responses, respectively (Figure 3). Most of the *PavGST* components were found to be involved in the appropriate expression of promoters, such as CAAT-Box (82%). Moreover, we discovered GRAS *cis*-elements relevant to plant growth/development, comprising 02-site of having 2%, which are linked to zein metabolic responsiveness (Li et al., 2020).

### Mode of Gene Duplications and Ka/ks Analysis in *PavGST* Genes

**FIGURE 4:**
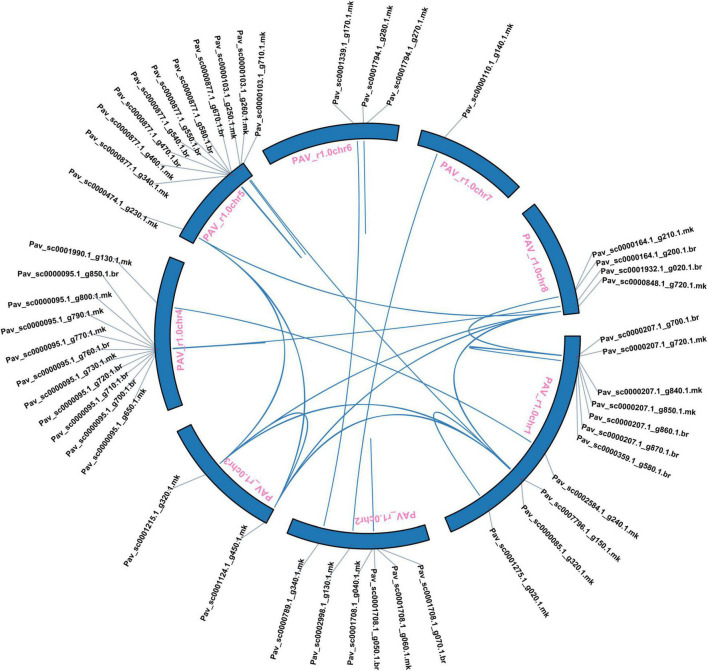
*Prunus avium* have chromosomal localization and mode of gene duplication. A colorful line links duplicated gene pairs.

**FIGURE 5:**
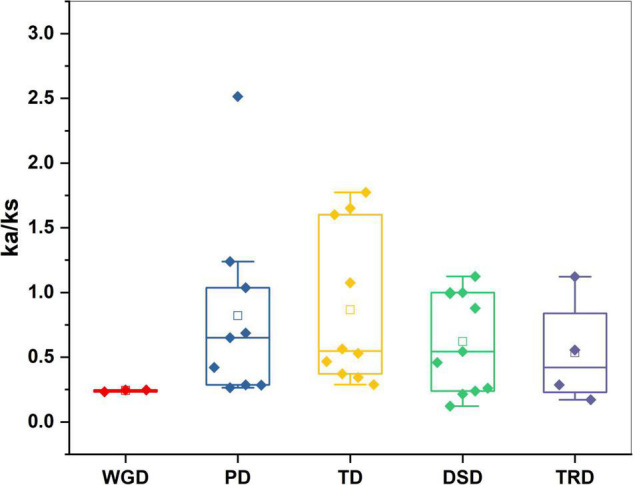
Ka/Ks values of GST genes family in *Prunus avium*. Comparison of Ka/Ks values for different modes of gene duplications. WGD, whole-genome duplicates; PD, proximal duplicates; TRD, transposed duplicates; TD, tandem duplicates; DSD, dispersed duplicates.

Five types of duplication whole genome duplication (WGD), proximal duplication (PD), tandem duplication (TD), dispersed duplication (DSD), and transposed duplication (TRD) were used to better understand the evolutionary history of the GST TFs gene family in sweet cherry. In sweet cherry, 37 pairs of genes were duplicated, along with WGD (three pairs), TRD (four pairs), DSD (11 pairs), TD (10 pairs), and PDs (9 pairs), indicating the gene family’s growth (Figure 4 and Supplementary Table 3). Dispersed duplication (DSD) event participation revealed its crucial role in the expansion of the GST family in sweet cherry. Furthermore, these results lead to the intricate duplication process of the GST family. All duplication mechanisms were incorporated in the development and expansion of *PavGST* genes (WGDs, DSDs, PDs, TDs, and TRDs). Dispersed duplication (DSD) was identified in 30% of sweet cherry genes, whereas tandem duplication (TD) was found in only 27%, suggesting that dispersed duplication events are more important in the expansion and evolution of the GST gene family than tandem duplication and other events. We estimate the evolutionary age of gene duplication events and selections by computing synonymous (Ks) and the non-synonymous (Ka) rates across all duplicated gene pairs. The results revealed that the Ka/Ks ratio ranged between 2.51 and 0.12 (Figure 5 and Supplementary Table 3). Positive selection was indicated by Ka/Ks ratios of more than 1, purifying selection by Ka/Ks values less than 1, and neutral selection by Ka/Ks = 1. Our study found that the majority of *PavGST* gene pairs had a Ka/Ks ratio smaller than 1, indicating that these genes are mostly exposed to purifying selection. On the other hand, the Ka/Ks ratio of two duplicated gene pairs is equal to one, indicating that neutral selection occurred (Figure 5 and Supplementary Table 3), but only nine *PavGST* gene pairs have a ka/ks ratio greater than one, showing that positive selection was dominated. Additionally, the Ka/Ks value was calculated in WGD, TRD, TD, PDs, and DSD. The highest Ka/Ks values were found in *Pav sc0000095.1 g790.1.mk* (Ka/Ks 2.51), which is situated on Chromosome 4, indicates a complicated evolutionary history for the GST gene family in sweet cherry.

### Chromosomal Localization and Synteny Analysis of *PavGST* Genes

**FIGURE 6:**
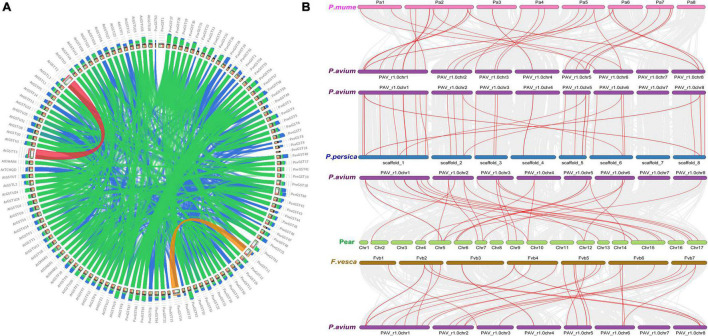
**(A)** Collinearity relationship among *Prunus avium* and *Arabidopsis thaliana*. **(B)** Collinearity relationship of GST genes in *Prunus avium* and other Rosaceae species (*Prunus persica, Prunus avium, Prunus mume*, and *Fragaria vesca*).

We further investigated the collinearity relationship of GST genes between *P. avium* (sweet cherry), *Pyrus bretschenedri*, (Chinese pear), *Fragaria vesca* (strawberry), *P. persica* (peach), and *P. mume* (Japanese apricot) since they all relate to the Rosaceae family and have a mutual ancestor (Figure 6 and Supplementary Table 4). There were 131 orthologous gene pairs identified among the Rosaceae genomes, comprising 30 orthologous gene pairs among sweet cherry and pear, 37 orthologous gene pairs between sweet cherry and peach, 31 orthologous gene pairs among sweet cherry and strawberry, and 33 orthologous gene pairs amid sweet cherry and apricot, implying a strong relationship among genomes of the Rosaceae species (Figure 6 and Supplementary Table 4). These results illustrated that the sweet cherry genome and the other Rosaceae genomes have a collinearity relationship, indicating a possible evolutionary relationship between them. Additionally, collinearity relationship in sweet cherry and pear, maximum orthologous pairs (10) were identified on Chr1 while chr4 had only two orthologous pairs. In all other Rosaceae species, such as sweet cherry and strawberry, Chr1 consists of 10 orthologous pairs, while minimum pairs (2) were identified on Chr4. On the other hand, in the sweet cherry and peach collinearity relationship, Chr1 contained a maximum of 12 pairs while Chr4 had only two orthologous pairs. Moreover, in sweet cherry and Japanese apricot, Chr1 expressed its dominancy and contained a maximum (11) pairs. These results demonstrated that Chr1 went under extreme evolutionary events while Chr4 faced minimum evolutionary events (Figure 6 and Supplementary Table 4).

**FIGURE 7:**
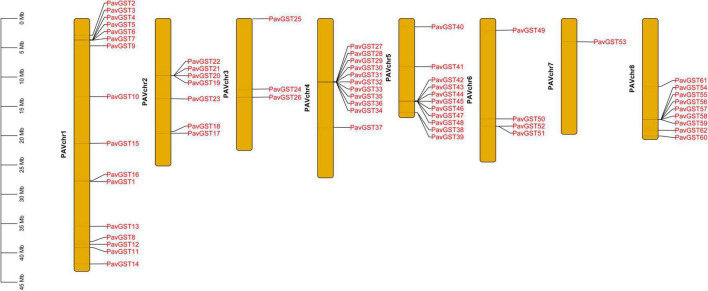
Chromosome localization of *PavGST* genes on 8 chromosomes and scale represents the length of each chromosome.

Subsequently, the chromosomal localization of GST genes in *P. avium* was also examined. 62 *PavGST* were identified on 8 chromosomes while 5 *PavGST* genes were located on the scaffold. The highest number of *PavGST* genes (16) was identified on chr1, while Chr7 had only one *PavGST* gene. Chr4 and Chr5 contained 11 *PavGST* genes in the form of clusters, while 7 *PavGST* members were localized on Chr2 in the cluster formation in the center of the chromosome. Chr3 and Chr6 had 3 and 4 *PavGST* genes, respectively, and all these GST members were identified in scattered formation in the whole chromosome (Figure 7 and Supplementary Table 1).

### Functional Annotation Study of *PavGST* Genes

**FIGURE 8:**
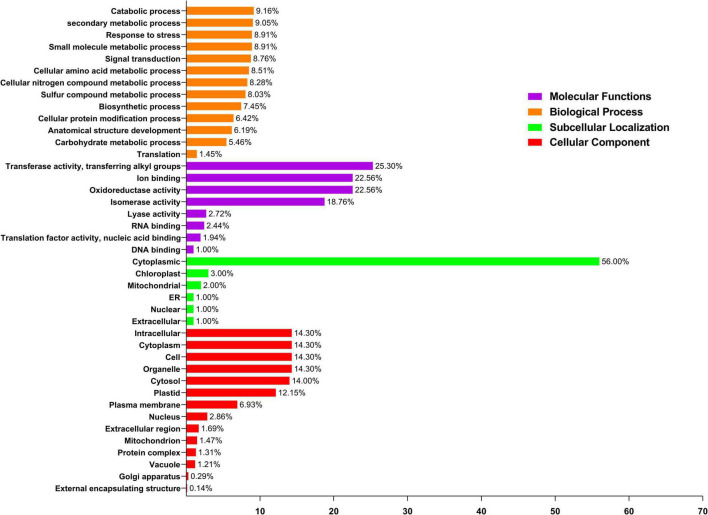
Gene ontology (GO) annotation of *PavGST* proteins. The GO annotation was performed based on three categories, Molecular function, cellular component, and biological process. The numbers on the bars represent the number of predicted proteins.

Gene ontology (GO) enrichment was evaluated in the biological process, subcellular localization, molecular function, and cellular components, which was beneficial for understanding protein function at the molecular level. As a result, the CELLO2GO tool was used to conduct a GO enrichment study of *PavGSTs* (Figure 8 and Supplementary Table 5). A total of 15 functional groups were found to be related to cellular components, 8 groups were traced with subcellular localization, eight groups were found to be engaged in molecular functions, and the other 13 groups might well be important in plant biological processes (Figure 8). In molecular functions, the function of ion binding, DNA binding, nucleic acid binding, isomerase activity, oxidoreductase activity, and transferase activity TFs were found in 22.56, 1, 1.94, 18.76, 22.56, and 25.30% of *PavGST*, respectively (Figure 8), indicating that these genes may control gene transcription and expression *via* these activities. On the other hand, the biological process GO term showed that *PavGST* participates in the catabolic process (9.16%), secondary metabolic process (9.05%), response to stress (8.91%), signal transduction (8.76%), and biosynthetic process (7.45%). Go ontology also revealed that *PavGST* genes are involved in the cellular protein modification process (6.42%), anatomical structure development, and carbohydrate metabolic process (5.46%). Furthermore, the cellular component GO term demonstrated that most of *PavGST* (14.30%) were found in the intracellular, cytoplasm, cell, and organelle, while plastid, plasma membrane, and nucleus had only 12.15, 6.93, and 2.86%, respectively. Finally, subcellular localization demonstrated that 56, 3, and 2% of *PavGST* are related to cytoplasmic, chloroplast, and mitochondrial, respectively, while only 1% of *PavGST* members were found in ER, nuclear, and extracellular (Figure 8).

### Transcriptional Profiling of Sweet Cherry Glutathione S-Transferases Genes

**FIGURE 9:**
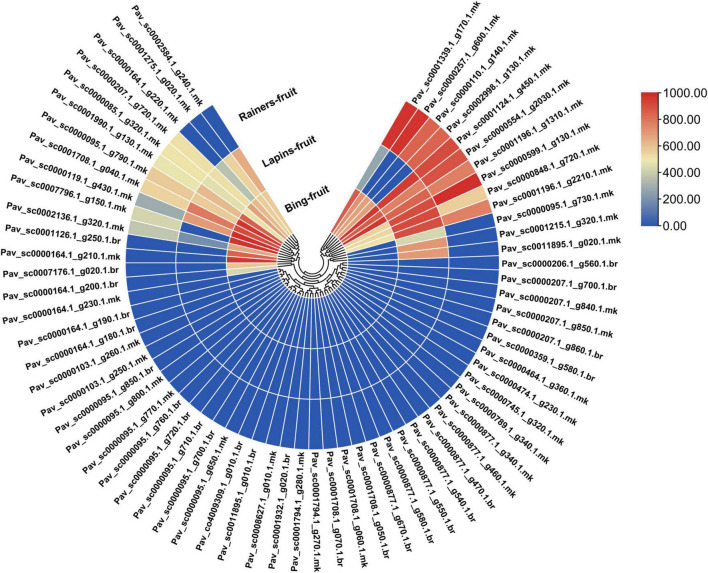
Transcriptomic evaluation of 67 *PavGST* genes in the fruit of three sweet cherry cultivars (Bing, Lapin, Rainer). Different colors revealed expression intensity.

GST is a critical transporter implicated in anthocyanin accumulation, according to several studies (Hu et al., 2016; Luo et al., 2018; Zhao et al., 2020). We utilized RNA-seq data from previous research to investigate the intimate link between *PavGSTs* and anthocyanin metabolic pathways. The gene expression was examined using fragments per kilo base million (FPKM) measurements. Transcriptomic data was used to analyze the expression patterns of *PavGST* genes in various cultivars (Bing, Rainers, and Lapins). Distinct cultivars exhibited different expression patterns for GSTs genes (Figure 9 and Supplementary Table 6). Based on expression patterns of *PavGST* members were classified into three groups (Supplementary Table 6). The first group was upregulated in all cultivars with 14 members (20.89%) in which some members were highly expressed in all stages like *Pav_sc0001196.1_g1310.1.mk* and *Pav_sc0001124.1_g450.1.mk* were extremely expressed in all cultivars while some members like *Pav_sc0000599.1_g130.1.mk* and *Pav_sc0001196.1_g2210.1.mk* and exhibited their extreme expression in specific cultivars like in Lapins and Rainers and expressed a little bit lower expression in Bing. These findings demonstrated that several *PavGST* members are cultivar-specific since they are strongly expressed in specific cultivars as compared to others. On other hand, the same phenomenon was identified in the second group; 14 members (20.89%) were only expressed in specific cultivars and remained silent in remaining cultivars like *Pav_sc0000164.1_g210.1.mk*, *Pav_sc0001126.1_g250.1.br*, *Pav_sc0007176.1_g020.1.br*, and *Pav_sc0000164.1_g200.1.br* expressed their expression in only in Bing but remain silent in other cultivars. The same was also noted in *Pav_sc0001215.1_g320.1.mk*, *Pav_sc0011895.1_g020.1.mk*, and *Pav_sc0000095.1_g730.1.mk*, which only exceedingly expressed in Lipins and remains silent in other cultivars. Different expression patterns indicated the diverse activities of *PavGST* genes in the respective pathways, hence offering a guide for identifying functional genes. The remaining 39 members (58.23% expression) have not been discovered in any cultivar and are remained silent (Figure 9 and Supplementary Table 6). These results revealed that these *PavGST* members might have some other particular functions in sweet cherry.

### Anthocyanin Content Accumulation

**FIGURE 10:**
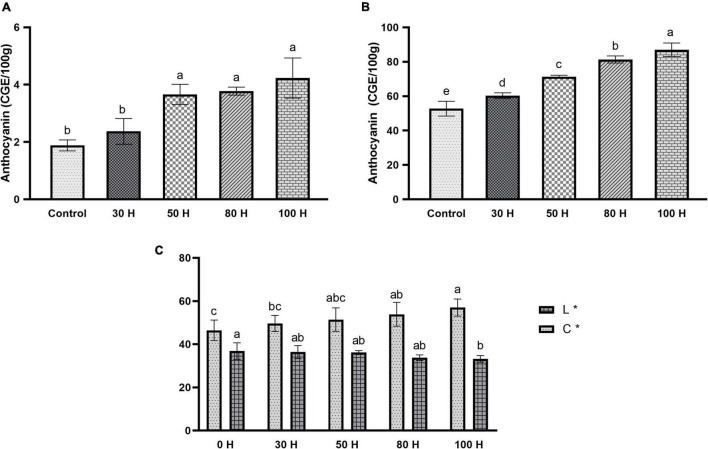
**(A)** Effect of LED light on anthocyanin contents in pulp (CGE/100 g) in sweet cherry cv. “Van.” **(B)** Effect of LED light on anthocyanin contents in peel CGE/100 g) in sweet cherry cv. “Van.” **(C)** Effect of LED light on color enhancement (L*, C*) sweet cherry cv. “Van.” Data are average ± SE. Different letters represent the significant difference between different time periods at 0.05 probability level (LSD test).

Anthocyanin is a key pigment in plants and getting more interest from researchers due to its extensive wide range of color, prominent beneficial health effect, and higher antioxidant activities. Moreover, we further measured the anthocyanin contents in the peal and flesh of sweet cherry fruit after applying the LED treatment for different time durations (30H, 50H, 80H, 100H). The results revealed the enhancement pattern for anthocyanin accumulation in the peel of the fruit when it was treated with LED light. As the duration of LED light increased the anthocyanin content also increased (Figure 10B). A significant difference was observed in all treatments as compared with control and also same results were identified between treatments (Figure 10B). Additionally, a slight change in anthocyanin contents was observed in the flesh of the fruit. All treatments demonstrated meaningful differences as compared with control except the 30H treatment. Maximum anthocyanin was noticed at 100H as compared with control (Figure 10A).

### Fruit Color and Antioxidant Evaluation

**FIGURE 11:**
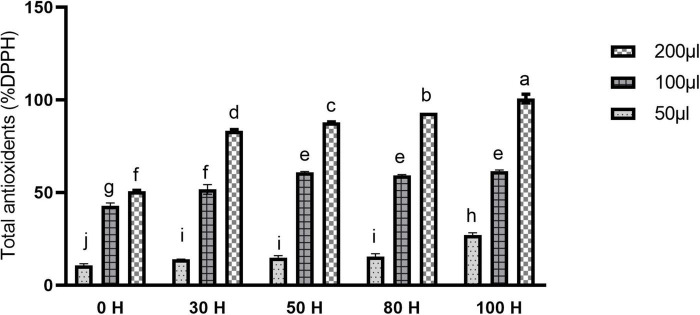
Effect of LED light on Total antioxidants (%DPPH) in sweet cherry cv. “Van.” Data are average ± SE. Different letters represent significant difference between different time periods at 0.05 probability level (LSD test).

Antioxidants perform a key role in health-enhancing biochemical pathways. Moreover, our results revealed the enhancement pattern in antioxidant contents. The scavenging activity of fruit extract against DPPH was concentration-dependent. Different concentrations expressed different free radical scavenging activity by DPPH. At 50 μlconcentration, maximum activity was found in 100H treatment as compared to control while other treatments also showed a significant difference as compared with control (Figure 11). However, using 200 μl concentrations, a significant difference was found in all treatments as compared to control, as well as between treatments also. Maximum activity was observed in the 100H treatment as compared with control while the minimum was noticed in the 30H treatment. However, other treatments also revealed significant differences as compared with control while at 100 μl concentration all treatments showed significant results as compared with control (Figure 11). Additionally, color intensity (C * value) and lightness (L * value) in sweet cherry “cv. Van” is shown in Figure 10C. Color saturation is displayed by the chroma index (C *). Higher values are denoted by the increment of intense red color.

### Expression Analysis of *PavGSTs* Through Quantitative Real-Time PCR

**FIGURE 12:**
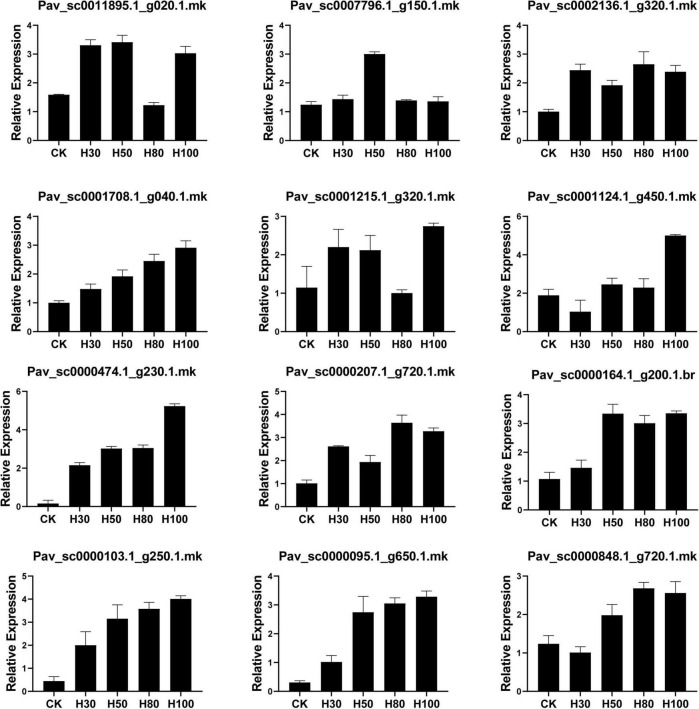
Relative expression behavior of *PavGST* genes through qRT-PCR on the different time duration of LED implementation on fruit. Mean ± SE of three biological replicates (each having three technical replicates).

Sun light is the most crucial factor for plant development and adequate phytochemical accumulation. However, due to continual gloomy, rainy days, greenhouse structures, and covers, the intensity of sunshine in the glasshouse is often insufficient for plants to develop properly and synthesize phytochemicals (Bian et al., 2015). In a regulated context, artificial LED light has been strategically implemented to enhance plant food quality, particularly the concentration of phenolic chemicals (Bian et al., 2015). Plant phytochemical concentration is affected by light quality (Kopsell and Kopsell, 2008). LED light has been shown to boost anthocyanin in tomatoes (Giliberto et al., 2005) and buckwheat seedlings (Azad et al., 2020). The GST gene family plays a vital role in anthocyanin accumulation (Fang et al., 2020). For validation, four LED treatments (30H, 50H, 80H, 100H) were applied to Van cultivar and were compared with the untreated control. 12 different *PavGST* transcripts belonging to different subclasses (DHAR, phi, tau, theta, and lambda) were selected and validated using qRT-PCR (Figure 12). For the Tau subclass, we found that *Pav_sc0000164.1_g200.1.br* (*PavGST56*) and *Pav_sc0011895.1_g020.1*.mk (*PavGST67*) were the most highly expressed in all four treatments as compared to control while *Pav_sc0002136.1_g320.1.mk* (*PavGST14*), *Pav_sc0001708.1_g040.1.mk* (*PavGST19*) and *Pav_sc0000207.1_g720.1.mk* (*PavGST4*) showed mild positive expression pattern as compared to untreated control. For the phi subclass, we found that two transcripts (*Pav_sc0001124.1_g450.1.mk (PavGST25), Pav_sc0000848.1_g720.1.mk (PavGST60)* were more highly expressed in the 80H and 100 H while *Pav_sc0001215.1_g320.1.mk (PavGST26)*, and *Pav_sc0007796.1_g150.1.mk (PavGST16)* expressed slightly positive expression pattern as compared to control (Figure 12).

Moreover, *Pav_sc0000474.1_g230.1.mk (PavGST40-Phi)*, *Pav_sc0000103.1_g250.1.mk (PavGST38-Tau) and Pav_sc0000095.1_g650.1.mk (PavGST27-Tau)* showed some interesting expression pattern, (Figure 12) which illustrated that these *PavGST* genes did not express in control treatment but after applying the LED light, all these three-member expressed extensive expression. RNA seq analysis also exhibited the silent behavior of these three genes in all cultivars. These results illustrated that these genes play a vital role in anthocyanin accumulation under the specific artificial LED light. The results also revealed that the GST gene family play a key role in anthocyanin accumulation in sweet cherry.

## Discussion

In the cytoplasm, anthocyanins are synthesized by flavonoid metabolic pathways and then transported to vacuoles for storage (Gu et al., 2019). Earlier research has demonstrated the intracellular transport mechanism for anthocyanin. GST mediation, vesicle trafficking, or membrane transport are all required for anthocyanins to infiltrate vacuoles from the cytoplasm (Zhao, 2015). GSTs are versatile enzymes that play a role in the production of secondary metabolites. The role of GSTs for anthocyanin accumulation has been investigated in many plants like Arabidopsis (Sun et al., 2012), strawberry (Lin et al., 2020), peach (Zhao et al., 2020), litchi (Hu et al., 2016), cyclamen (Kitamura et al., 2012), apple, and pear (Zhao et al., 2021). GST is a supergene family found in higher plants that are subdivided into subclasses F, U, Z, T, L, EF1B, GHR, DHAR, and TCHQD (Nianiou-Obeidat et al., 2017). Numerous GSTs have been identified in plants so far, including 64 GSTs in Arabidopsis (Sappl et al., 2009), 52 GSTs in apple (Zhao et al., 2021), 82 GSTs in radish (Gao et al., 2020), 42 GSTs in pear (Zhao et al., 2021), 139 GSTs in litchi (Hu et al., 2016), 50 GSTs in peach (Zhao et al., 2020), and 29 GSTs in strawberry (Luo et al., 2018). GSTs have been found in a variety of plant species in recent years, and the number and content of GST family members vary across species. Nevertheless, no genome-wide identification and characterization of the GST gene family in *P. avium* has been revealed.

Our research offers a comprehensive and systematic whole-genome evolutionary investigation of GST (Vaish et al., 2018) members derived from sweet cherry genomes. There were a total of 67 GST genes found in the *P. avium* (Supplementary Table 1). Our phylogenetic findings suggest that sweet cherry genomes have undergone gene acquisition. *PavGST* genes were found to be present in each of the seven main GST subfamilies (Tau, DHAR, Lambda, Zeta, Phl, Theta, and TCHQD) that were identified by phylogenetic analysis (Figure 1) and concluded to be identical to other plant species which were studied previously (Csiszár et al., 2014; Islam et al., 2018; Wang et al., 2018). The Tau subfamily contained the highest *PavGST* member. The same phenomenon was also confirmed in apples (Fang et al., 2020). Moreover, the number of amino acids of GST proteins ranged from 61 to 527 kDa with an average of 219.42kDa (Supplementary Table 1) which revealed a significant variation among *PavGST* members. However, the differences across clades might be associated with the different functionalities and diversity of conserved motif structure and exons/introns. The properties of GSTs’ gene structure were retained in apple, Populus, sweet, and potato (Lan et al., 2009; Ding et al., 2017; Islam et al., 2018). It is also important to note that intron and exon variants play a crucial influence in gene evolution (Xu et al., 2011; Jo and Choi, 2015; Mustafin and Khusnutdinova, 2015). The 67 GST genes in this research have varying numbers of introns and exons, suggesting the heterogeneity of sweet cherry GST genes. The *PavGST* genes had varied intron numbers from 1 (*PavGST29, PavGST31, PavGST10*) to 12 (*PavGST11*) while exon numbers varied from 1 (*PavGST54, PavGST8*, and *PavGST39*) – 10 (*PavGST53* and *PavGST64*; Figure 2). Additionally, the majority of the genes in the same subfamily exhibited comparable motif compositions. A total of 20 distinct motifs were discovered among the GST genes of the *P. avium* and at least one of these is present in each candidate gene. The arrangement and quantity of the 20 different intra-species and inter-species motif types revealed that GST members are functionally diverse (Figure 2). Moreover, the protein functions are thought to be related to its subcellular localization (Dönnes and Höglund, 2004). The function of a protein is thought to be related to its subcellular localization (Dönnes and Höglund, 2004). The bioinformatics examination in this work revealed that the majority of GSTs were found in the cytoplasm (Figure 8 and Supplementary Table 5), which was similar to prior research (Dong et al., 2016; Islam et al., 2017; Kayum et al., 2018). GSTs involved in flavonoid transport have been linked to membranes, including the vacuole and the endoplasmic reticulum (ER; Conn et al., 2008; Kitamura et al., 2010; Gomez et al., 2011; Zhao, 2015). GSTs involved in flavonoid transport have been linked to membranes, including the endoplasmic reticulum (ER) and the vacuole (Conn et al., 2008; Gomez et al., 2011; Zhao, 2015). This divergence might be attributable to a program limitation or the complexities of protein localization (Xiong et al., 2016). Gene duplications are an important process in all plants for producing genetic innovation, which might assist organisms in adapting to environmental change (Alvarez-Buylla et al., 2000; Kondrashov, 2012). Different types of gene duplication, such as TD, PD, DSD, WGD, and TD, significantly contribute to plant-specific gene expansion (Qiao et al., 2018; Si et al., 2019). Typically, whole-genome duplication (WGD), segmental duplication, and tandem duplication events are regarded to be the key driving factor for producing new genes and gene family expansion (Rensing, 2014). We observed that TD and DSD contributed significantly to GST family member expansions in sweet cherry (Figure 4 and Supplementary Table 3). The same phenomenon was observed in apple, rice, *G. Arboreum*, *G. raimondii* (Dong et al., 2016), poplar (Lan et al., 2009), and *Capsella rubella* (He et al., 2016). Gene duplication is crucial not just for expanding genomic content, but also for producing novel gene functions, which may help organisms to adapt to complicated surroundings (Freeling, 2009). In addition, we used CDS to examine the frequencies of ka and ks substitutions (coding sequences). Positive selection is defined as a value larger than 1, purifying selection as a value less than 1, and neutral selection as a value of 1 (Volokita et al., 2011; Zhang et al., 2014). All Ka/Ks ratios of paralogous genes show that GST protein activities may be predominantly determined by purifying selection (Figure 5 and Supplementary Table 3). Mode of duplication events and the ages of genome duplication events show that GSTs evolved through a complex mechanism. The addition or deletion of an exon or intron is critical for the diversification of multigene families. This event might occur as a result of the rearrangement and fusion of several chromosomal segments (Xu et al., 2012).

Light is one of the most important components in plant growth and development since it directly regulates plant morphogenesis, gene expression, metabolism, photosynthesis, and other physiological reactions. Light conditions can alter qualitative and quantitative changes in anthocyanin. Numerous research has been conducted in the past decade to examine the effects of light quality and intensity on plant development and functional chemical yields (Goto, 2012). Anthocyanin is a key pigment in plants and attracting more interest from researchers due to its extensive and wide range of color, prominent beneficial health effect, and higher antioxidant activities. They are also involved in antimicrobial activity and protect the cells from intensive light damage by absorbing blue and ultraviolet light (Seigler, 1998). Insufficient light and nutrient availability decreased the anthocyanin contents in fruits (Youdim et al., 2002; Ohashi-Kaneko et al., 2007). In the current study, led light was applied to the fully mature fruit of “Van” sweet cherry cultivar for different time durations (30H, 50H, 80H, and 100H). The results illustrated the boosted pattern in anthocyanin accumulation in fruit skin and pulp (Figure 10). The same enhancement pattern was observed in strawberries and grapes when treated with LED light (Kim et al., 2005; Kadomura-Ishikawa et al., 2013; Kondo et al., 2014). LED application for a sufficient period may be an effective technique for increasing plant anthocyanin content (Azad et al., 2020). The molecular processes that underlie anthocyanin accumulation caused by LEDs are yet not completely known. Previous research has shown that LED stimulates the expression of anthocyanin biosyntheses structural genes, such as PHYs and CRY, as well as regulatory genes, such as MYB, bHLH, and WRKY (Zhang et al., 2018). Moreover, antioxidants perform a key role in health-enhancing biochemical pathways (Hogewoning et al., 2010) and bioactive compounds like carotenoids, flavonoids, and phenolic which have potential for antioxidant activity (Fernández-Ruiz et al., 2017). LED light also placed a positive impact on antioxidant accumulation in sweet cherry, as well as same enhancement pattern of antioxidant contents was confirmed in Chinese kale sprouts and fruit when treated with blue LED light as compared with control (Galuszka et al., 2005; Qian et al., 2016). Additionally, we also investigated and confirmed transcriptomic data obtained from anthocyanin accumulation to acquire insight into the expression patterns of GSTs in response to the accumulation of anthocyanin using qRT-PCR (Figure 12). In response to LED treatments, a distinct divergence in the expression patterns of *PavGSTs* was identified among them. Based on the qRT-PCR analysis, Tau members, such as *Pav sc0000164.1 g200.1.br* (*PavGST56*), *Pav sc0000207.1 g720.1.mk* (*PavGST4*), and *Pav sc0011895.1 g020.1.mk* (*PavGST67*), reacted swiftly to LED treatment and exhibited high anthocyanin accumulation. Previous findings also demonstrated that Tau GSTs are involved in the detoxification of xenobiotics, the transmission of signals, and the transport of anthocyanin (Loyall et al., 2000; Thom et al., 2002; Jha et al., 2011). Moreover, two members from the Tau subfamily (*Pav_sc0000474.1_g230.1.mk -PavGST40, Pav_sc0000103.1_g250.1.mk -PavGST38*) and one from the Phi subfamily (*Pav_sc0000474.1_g230.1.mk* – *PavGST40*) only revealed their peak expression under higher LED light treatment which demonstrated that transcription of these *PavGST* genes is under the control of light. The same phenomenon was also confirmed in *Lilium regale* (Yamagishi, 2016). The RNA-seq data was carried out on the fruit of three sweet cherry cultivars: Bing, Lapins, and Rainers (Figure 9). These data were used to investigate the spatiotemporal expression profile of GST genes in different cultivars. *Pav_sc0001196.1_g1310.1.mk* and *Pav_sc0001124.1_g450.1.mk* genes were abundantly expressed in all cultivars, suggesting that these genes play a key role in anthocyanin accumulation. Additionally, *cis*-acting elements were critical in regulating the expression of genes and hence influencing plant responses to stress and developmental changes (Narusaka et al., 2003). The *cis*-regulatory elements analysis revealed that GST genes consist of abiotic/biotic stress-related elements and expression of promoters, including CAAT-Box, ABR, and LTR.

## Conclusion

The GST gene family has been identified in a wide variety of plant species and has been associated with several key physiological and developmental activities. In this investigation, 67 GST genes were identified in sweet cherry utilizing genome-wide analysis and grouped into seven subfamilies (TCHQD, Theta, Phi, Zeta, Lambda, DHAR, and Tau). The phylogenetic relationship, gene duplication events, conserved motif composition, collinearity analysis, cis-acting elements, conserved domain, chromosomal localizations, non-synonymous, synonymous ratios, and functional divergence of these *PavGST* genes were all investigated in detail, providing insight into the functional diversity of GST families. Tissue-specific expression and abiotic stress response studies revealed that the expression patterns of GST genes in sweet cherry are very specialized and diverse. The GST genes were highly upregulated in response to an LED treatment, showing that they play a critical role in the accumulation of anthocyanin in sweet cherries. In the future, this work can be used as a good benchmark for future functional studies and genetic breeding of GST genes in sweet cherry, as well as for the identification of relevant candidate genes for future anthocyanin accumulation studies.

## Data Availability Statement

The datasets presented in this study can be found in online repositories. The names of the repository/repositories and accession number(s) can be found in the article/Supplementary Material.

## Author Contributions

IS and MM conceived and designed the experiments. IS, MM, IHS, XL, SJ, JW, PA, and MA contributed reagents, materials, and analysis tools. CZ provided guidance on the whole manuscript. All authors read and approved the final manuscript.

## Conflict of Interest

The authors declare that the research was conducted in the absence of any commercial or financial relationships that could be construed as a potential conflict of interest.

## Publisher’s Note

All claims expressed in this article are solely those of the authors and do not necessarily represent those of their affiliated organizations, or those of the publisher, the editors and the reviewers. Any product that may be evaluated in this article, or claim that may be made by its manufacturer, is not guaranteed or endorsed by the publisher.

## Footnotes

1
https://www.rosaceae.org

2
http://www.ebi.ac.uk/interpro/

3
https://www.genscript.com/tools/pcr-primers-designer

4
http://gsds.gao-lab.org/

5
https://meme-suite.org/meme/tools/meme

6
https://mapinspect.software.informer.com/

7
https://bioinformatics.psb.ugent.be/webtools/plantcare/html/

8
https://popgenie.org/node/42

9
http://services.cbu.uib.no/tools/kaks

10
http://gsds.cbi.pku.edu.cn/

11
https://www.ncbi.nlm.nih.gov/Structure/lexington/lexington.cgi

12
http://hmmer.org

13
http://bioinformatics.psb.ugent.be/webtools/plantcare

14
http://chibba.agtec.uga.edu/duplication

15
https://github.com/qiaoxin/Scripts_for_GB/tree/master/calculate_Ka_Ks_pipeline

16
http://gsds.cbi.pku.edu.cn/index.php
